# Regeneration of Genetically Stable Plants from in Vitro Vitrified Leaves of Different Carnation Cultivars

**DOI:** 10.3390/plants9080950

**Published:** 2020-07-28

**Authors:** Ho Thi Minh Thu, Aung Htay Naing, Hui Yeong Jeong, Chang Kil Kim

**Affiliations:** Department of Horticulture, Kyungpook National University, Daegu 41566, Korea; thumiu.luv@gmail.com (H.T.M.T.); dmg03062@naver.com (H.Y.J.)

**Keywords:** carnation, in vitro regeneration, plant growth regulators (PGRs), RAPD, vitrified leaves

## Abstract

This study was conducted to investigate the efficacy of shoot regeneration from different leaf types (normal leaves and vitrified leaves) from three different carnation cultivars ‘Kumbuyl’, ‘Denev’, and ‘Jinju’ using different combinations of 3-indole butyric acid (IBA) and thidiazuron (TDZ) concentrations. The shoot tips cultured on Murashige and Skoog (MS) basal media (Type 1 media) produced normal leaves, while those cultured-on media supplemented with plant growth regulators and/or vitamin (Type 2 media and Type 3 media) produced vitrified leaves for all cultivars. Culture of normal leaf segments on MS medium containing different combinations of IBA and TDZ concentrations induced callus in all treatments; however, the callus was unable to induce shoots and finally became necrotic. In contrast, no callus induction was observed in the control (hormone-free treatment). When vitrified leaf segments underwent the same treatments, shoots were induced from the vitrified leaves (derived from Type 2 media) but were unhealthy and gradually died, whereas those induced from Type 3 media were vitrified and healthy. The optimal combination for the best shoot regeneration and number of shoots per explants varied depending on the genotypes used. The vitrified shoots induced from the leaves of Type 3 media transformed into normal shoots and survived well under greenhouse conditions. According to the results of random amplified polymorphic DNA (RAPD) analysis, the banding patterns of twelve primers that were detected in vitrified leaf-induced normalized shoots were identical to those of normal in vitro grown plants, indicating that no genetic variation had occurred during the procedure. Taken together, this study indicates that vitrified leaves can be used for shoot regeneration of recalcitrant carnation cultivars, regardless of the genotypes and types of vitrified leaves. However, as the number of shoots per explants was still low, further investigation is warranted to obtain a more efficient shoot regeneration protocol for genetic transformation of the cultivars.

## 1. Introduction

Carnations (*Dianthus caryophyllus* L.) are widely used as cut flowers, bedding plants, and pot plants and are ranked as the third-most important cut flowers after roses and chrysanthemums in the global floriculture industry owing to their diverse flower color, ability to withstand long distance transportation, and remarkable ability to rehydrate after continuous shipping. The demand for new carnation cultivars with novel traits has been steadily increasing in the global floricultural industry for several decades [1]. In response to this demand, plant biotechnologists have been creating new carnation cultivars with novel ornamental and agronomic traits using *Agrobacterium*-mediated genetic transformation [2,3,4,5,6,7]. In fact, an efficient in vitro shoot regeneration method is a prerequisite for the success of genetic transformation studies. In vitro regeneration protocols have been developed for a range of, but not all, carnation cultivars [8,9,10]. Generally, in vitro shoot regeneration of carnations is influenced by plant growth regulators (PGRs), explant types, and genotypes [11].

The prominent problem encountered during in vitro regeneration of carnation is vitrification, also known as hyperhydricity, because regenerated plants derived from vitrification exhibit physiological and morphological malformations [12,13,14]. Their stems are not only rigid and easily breakable but their leaves also contain low chlorophyll content and high water content; therefore, they cannot be easily transformed to normal plants [14,15,16]. However, in our preliminary experiments, leaves from such vitrified plants were able to induce shoots, whereas those derived from in vitro normal plants were unable to do so, although we did not investigate the genetic fidelity of the vitrified leaf-induced shoots in this experiment. Firoozabady et al. (1995) [17] also reported that vitrified leaves were able to regenerate shoots when they were used as explants for *Agrobacterium*-mediated genetic transformation in carnations. Therefore, in this study, we attempted to induce normal and vitrified leaves from three different carnation cultivars using three different media compositions and evaluated shoot induction efficiencies from the resulting normal and vitrified leaves using different combinations of PGRs. In addition, the genetic fidelity of the regenerated plants was determined using random amplified polymorphic DNA (RAPD) markers.

## 2. Results and Discussion

Despite several reports describing successful plant regeneration in carnations, its regeneration efficiency largely depends on explant types and ages, PGRs, and genotypes [18,19,20], suggesting that a protocol that is suitable for one cultivar is not applicable for other cultivars. Therefore, it is necessary to develop specific regeneration protocols for specific cultivars. The prominent problem encountered during in vitro regeneration of carnations is the occurrence of vitrification, resulting in vitrified shoots that cannot easily be transformed to normal and healthy shoots. To address this problem, research to reduce vitrification by optimizing several factors affecting vitrification has been undertaken [21,22]. However, to date, no regeneration protocol has been developed for the carnation cultivars KB, DNV, and Jinju used in this work. Therefore, we investigated protocols for in vitro shoot regeneration to be used for genetic transformation of these cultivars.

**Figure 1:**
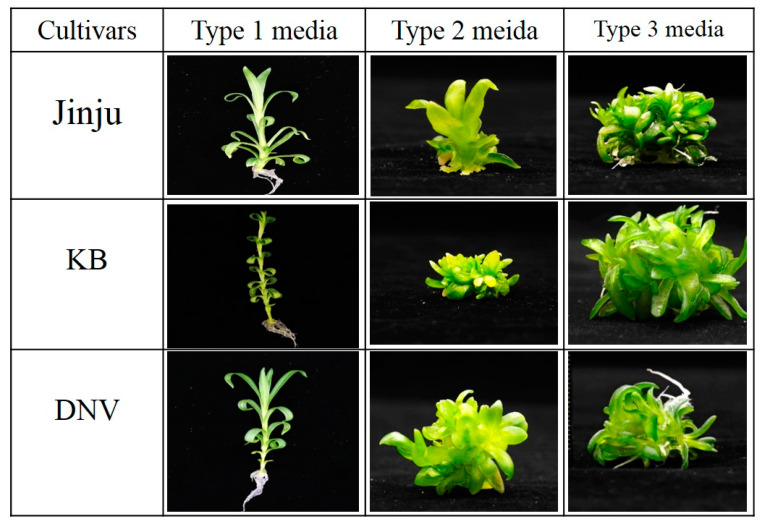
Effect of different types of media on the induction of normal and vitrified shoots in different carnation cultivars.

According to the results of our preliminary work, shoot regeneration was not possible when normal leaves were used as explants; however, several shoots were regenerated from vitrified leaf segments even when the same PGR concentration was used. Therefore, in this study, we cultured the shoot tips on three different kinds of media (namely Type 1, Type 2, and Type 3 media). Type 1 medium was prepared without PGRs to induce normal shoots, Type 2 medium was prepared with a high concentration of cytokinins, and Type 3 medium was prepared with adenine for the induction of vitrified shoots [17]. As expected, we obtained normal and vitrified shoots from the respective media (Figure 1). The stems of vitrified plantlets derived from Type 2 and Type 3 media were short, broad, and thick in diameter, whereas the leaves were greenish-yellow, thick, broad, wrinkled, and breakable. On the other hand, the plantlets derived using Type 1 medium were approximately 4–5 cm in height, and their leaves were greener, slender, and 2–3 cm long in length. Moreover, plants grown on Type 1 medium were not able to produce adventurous shoots compared with those grown on Type 2 or Type 3 media; this result was because the latter two media contained cytokinins or vitamins that promote shoot induction in carnations [23,24,25].

**Figure 2:**
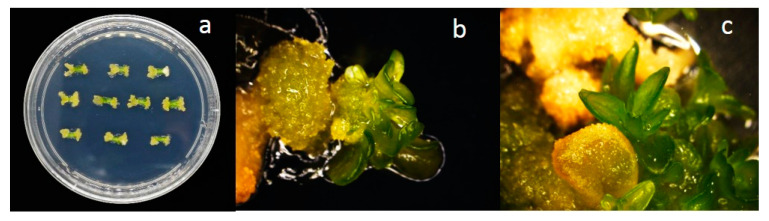
Panels showing different morphogenesis in carnation cv. ‘Jinju’ depending on the type of leaves used. (**a**) Callus induction from normal leaves (derived from Type 1 medium), (**b**) shoot regeneration from vitrified leaves (derived from Type 2 medium), and (**c**) shoot regeneration from vitrified leaves (derived from Type 3 medium).

When the leaf segments from normal leaves (derived from Type 1 medium) were cultured on the media containing different concentrations of IBA and TDZ combinations, callus induction started from the cut edges of the leaf explants within 7–9 days after culture (Figure 2a). Despite variation in the percentage of callus induction depending on genotypes and PGR combinations, callus induction was observed in all treatments for all cultivars. However, no callus induction was observed in the hormone-free MS media (control). Although the normal leaf-induced callus exhibited rooting, they did not convert to shoots and finally became necrotic. These results were not consistent with those of previous studies that succeeded in regenerating shoots from leaf-derived callus in carnations [18,19,20]. TDZ is known to be an effective cytokinin for regenerating shoots in carnation cultivars [26] and is more powerful than BA, kinetin, and zeatin for shoot regeneration in carnations. The failure of shoot regeneration in our study is likely due to differences in genotypes used in our studies.

When segments of vitrified leaf derived from Type 2 medium were cultured on the same treatments, shoot regeneration was obtained in all treatments, except control, for all cultivars. However, the percentage of shoot regeneration and number of shoots per explant were relatively low (data not shown). In addition, the regenerated shoots tended to be unhealthy, with glassier and greenish-yellow leaves (Figure 2b). This may be because the shoots derived from Type 2 medium were originally poor in quality (Figure 1); therefore, when using their leaves as explants, it was difficult to regenerate healthy shoots. In contrast to the leaves derived from Type 1 and 2 media, when the leaf segments derived from Type 3 medium were cultured on the same treatments, callus induction and shoot regeneration from the base of the leaf segments were observed at the same time after two weeks of culture. However, they were directly regenerated from the base of the leaf segments and were likely to be healthier than those obtained from the leaves of Type 2 medium-regenerated shoots (Figure 2c). Differences in shoot health obtained from leaves using Type 2 medium and those using Type 3 medium could be attributed to the differences in health of the donor plants, with Type 3 medium-derived shoots being in better condition than Type 2 medium-derived shoots. The regeneration of shoots from the base of the leaf segments could be attributed to the cells near the connecting zone between the leaf base and the node having a greater potential to regenerate shoots [23].

Among the combinations tested, the combination of 0.1 mg/L IBA and 0.2 mg/L TDZ was found to be the best for the percentage of shoot regeneration (66.67%) and number of shoots per explant (3.46) in cv. Jinju. Concentrations higher than these resulted in a rapid decline in the percentage of shoot regeneration and/or number of shoots per explant (Table 1). Similar results were also observed in cv. KB using the same combination (0.1 mg/L IBA and 0.2 mg/L TDZ) that produced the highest shoot regeneration percentage (26.67%) and a reasonable number of shoots per explant (2.90), whereas higher concentrations produced a lower number of shoots (Table 1). In contrast, even with the same optimal combination, the percentage of shoot regeneration and number of shoots per explants were lower in cv. KB than in cv. Jinju. However, for cv. DNV, the combination of 1.0 mg/L IBA and 0.2 mg/L TDZ gave the highest number of shoots per explant (3.83) but the percentage of shoot regeneration was only 16.67%. A combination of 0.5 mg/L IBA and 0.3 mg/L TDZ, resulted in a higher percentage of shoot regeneration (26.67%), but the number of shoots per explant was 2.83 (Table 1). Overall, PGR combinations require further optimization depending on the genotypes used because their optimal combination significantly affects the percentage of shoot percentage and number of shoots per explant. In general, the percentage of shoot regeneration and number of shoots per explant observed in this study was unsatisfactory for all cultivars. However, TDZ has been previously shown to be more effective than either BA or kinetin for shoot regeneration in carnations [27].

**Figure 3:**
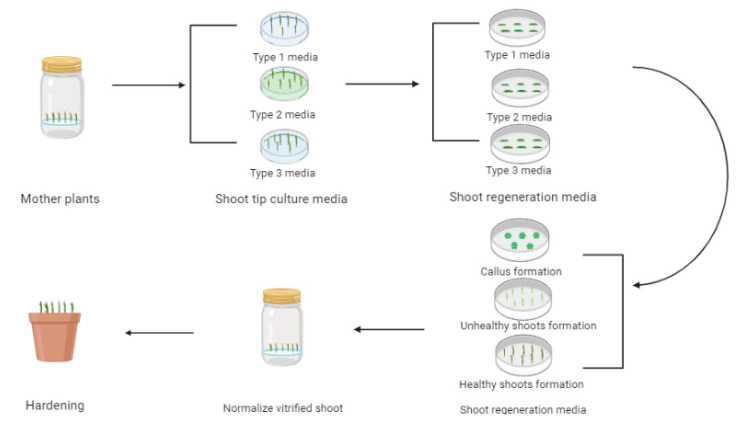
Flow chart showing efficacy of shoot regeneration from different types of leaves in the carnation cultivars.

The combination of 0.5 mg/L IBA and 0.2 mg/L TDZ has been previously shown to result in a higher percentage of shoot regeneration (88.6% and 100%) with other carnation cultivars [17,28], respectively), although this exhibited relatively lower results in our study. This result suggests that optimal concentration and/or combination is highly influenced by genotypes. Taken together, vitrified leaves derived from Type 3 medium were more efficacious for shoot regeneration from all cultivars in comparison with those derived from Type 1 and Type 2 media (Figure 3). However, as the percentage of shoot regeneration and number of shoots per explant were still low, continued optimization of PGR combinations is required to improve the results.

**Figure 4:**
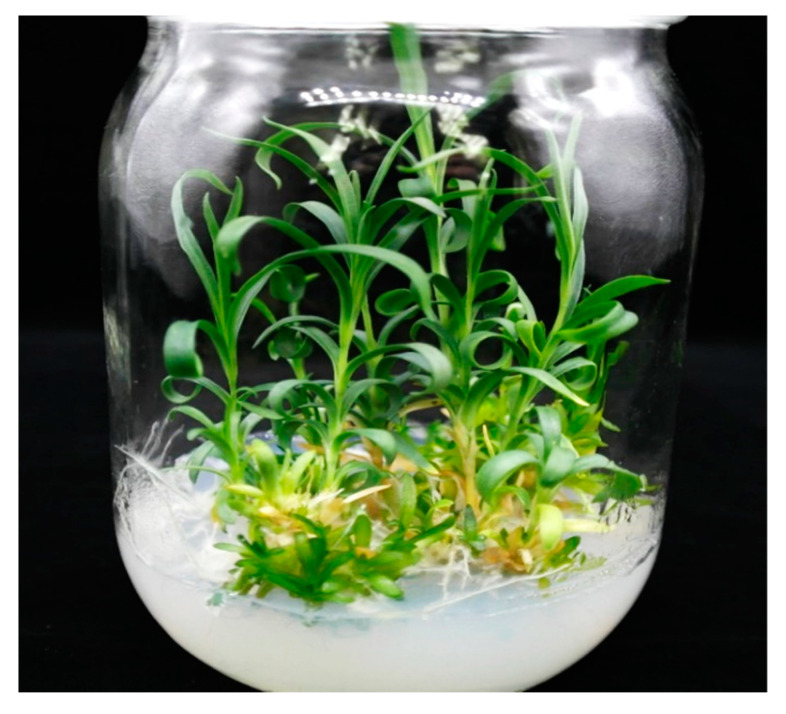
Conversion of vitrified plants to normal plants on the rooting and plant growth medium containing 1% plant agar.

Most of the shoots obtained in these above experiments were vitrified, and generally, they do not survive during acclimatization due to low chlorophyll contents in their leaves, glassiness, leaf curliness, and poor root induction; which finally turned to malformed plantlets. To address this issue, alterations in growing conditions have been investigated by adjusting the gaseous exchange in a container, concentrations of agar, and light density. In this study, when the vitrified shoots were transferred to hormone-free MS media containing 1.0% plant agar, the morphology of the vitrified shoots appeared normal: the leaves were a deeper green, less glassy, and not wrinkled, and the lengths of the internodes were longer (Figure 4). Subsequent transfer (at least two or three times) of the normalized plants to the same media containing 1.0% plant agar provided completely normalized plants with roots. Successful normalization of vitrified plantlets using a higher concentration of plant agar has also been reported [17].

**Figure 5:**
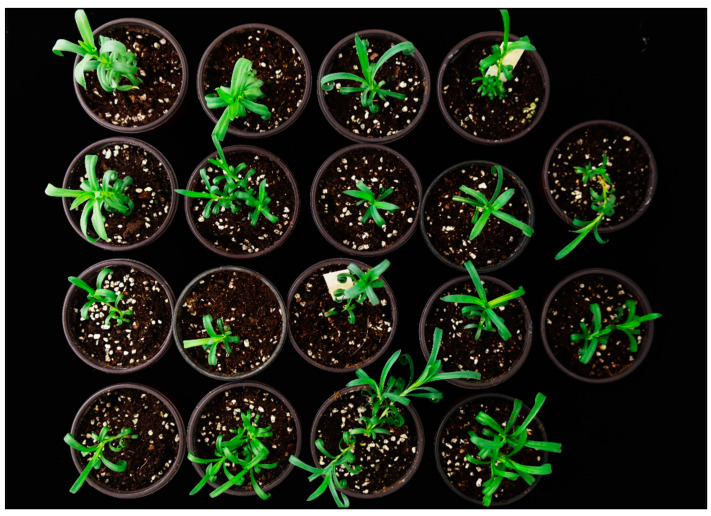
Vitrified-derived leaves in vitro regenerated, and normalized plants of the carnation cultivars then survived well under greenhouse conditions.

Before transplanting to the greenhouse, the normalized plants from all cultivars were transplanted to small pots containing peat-based soil and were acclimatized in the growth chamber for about one week, as hardening the plantlets at the transitions from in vitro to ex vitro is necessary to increase the survival of transplants [29]. In our study, all plantlets were observed to survive during the acclimatization period and maintained their survival rate when transferred to the greenhouse (Figure 5).

**Figure 6:**
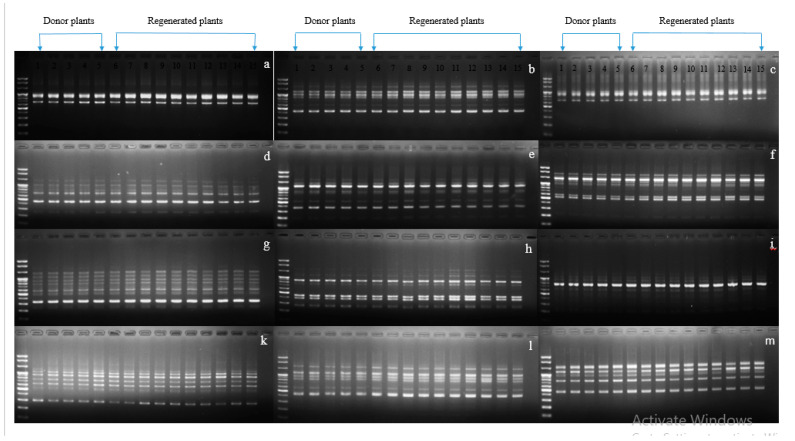
Showing genetic stability between vitrified-derived plants and donor plants using random amplified polymorphic DNA (RAPD) markers in cultivar Kumbuyl (KB). (**a**) OPA 03, (**b**) OPA 04, (**c**) OPA 07, (**d**) OPA 10, (**e**) OPA 19, (**f**) OPB 01, (**g**) OPB 17, (**h**) OPA 01, (**i**) OPA 02, (**k**) OPB 06, (**l**) OPB 07, (**m**) OPB 10.

**Figure 7:**
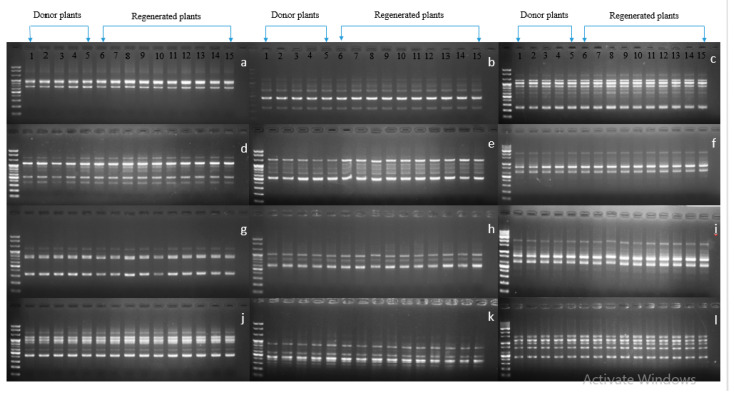
Showing genetic stability between vitrified-derived plants and donor plants using RAPD markers in cultivar Denev (DNV). (**a**) OPA 03, (**b**) OPA 07, (**c**) OPA 10, (**d**) OPA 18, (**e**) OPA 19, (**f**) OPA 20, (**g**) OPB 12, (**h**) OPB 17, (**i**) OPB 18, (j) OPA 09, (**k**) OPB 07, (**l**) OPB 10.

**Figure 8:**
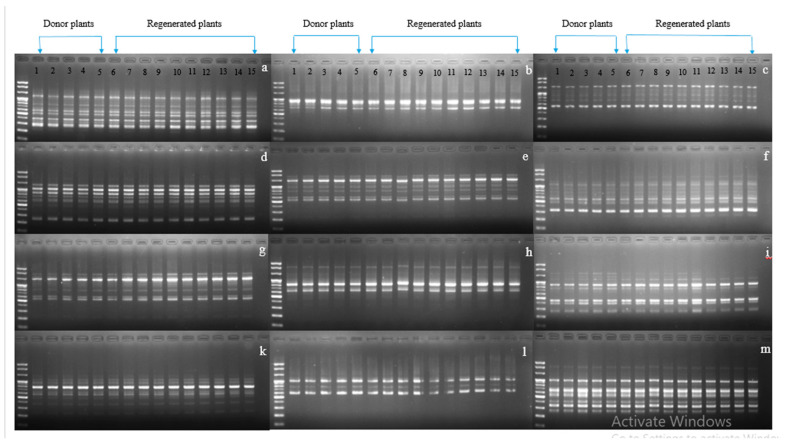
Showing genetic stability between vitrified-derived plants and donor plants using RAPD markers in cultivar Jinju. (**a**) OPA 04, (**b**) OPA 07, (**c**) OPA 20, (**d**) OPA 18, (**e**) OPA 19, (**f**) OPB 01, (**g**) OPB 11, (**h**) OPB 17, (**i**) OPA 01, (**k**) OPA 02, (**l**) OPB 18, (**m**) OPB 10.

Genetic variation may occur through several genetic mechanisms (e.g., point mutations, DNA methylation, and structural chromosomal changes). Detection of genetic stability in in vitro regenerated carnations and other horticultural crops has been detected using RAPD markers [1,30,31,32]. In our study, because the normalized plants originated from the leaves of vitrified plants, it was interesting to investigate whether they were genetically stable or not; therefore, the stability of the normalized plants along with in vitro grown normal plants were assessed for all cultivars using RAPD markers with 40 primers. Twelve sets of primers were shown to be scorable, and their banding patterns detected in normalized plants were identical to those of in vitro grown normal plants, indicating there had been no genetic variation among the tested plants (Figure 6, Figure 7 and Figure 8). This result was consistent with that of Aalifar et al. [33], who recently reported that there was no genetic variation between in vitro regenerated shoots and mother plants.

Overall, the leaves of vitrified plants harbor a comparatively greater potential to regenerate shoots than those of normal plants. Although the regenerated shoots in our study were vitrified and appeared to be malformed, we did not observe genetic variation in the plants normalized from the vitrified plants. In fact, only because of the media composition used, the resulting shoots were to be vitrified and deficient in physiological condition, but no genetic variation was observed in the vitrified plants (non-normalized plants) when evaluating those using RAPD markers (data not shown). Therefore, no genetic variation was observed in the normalized plants that were derived from the leaves of vitrified plants compared with in vitro grown normal plants. Our findings suggest that in vitro vitrified plants need to be produced for the recalcitrant carnation cultivars in which normal leaves are unable to multiply shoots, and the vitrified leaves can be used as an explant source for in vitro regeneration.

## 3. Conclusions

In this study, we investigated shoot regeneration from three different carnation cultivars, namely Denev, Jinju, and Kumbuyl, using different combinations of IBA and TDZ concentrations and different sources of leaves (normal and vitrified leaves). Shoot regeneration was not successful from normal leaves (derived from Type 1 medium) even when cultured on the media containing various combinations of PGRs, and only callus formation was observed in all cultivars. When culturing vitrified leaves (derived from Type 2 medium) on the same PGR treatments, shoot induction was observed in all cultivars, but these were likely to be unhealthy and gradually died. In contrast, vitrified leaf segments derived from Type 3 medium were able to induce shoots that were healthier than those derived from Type 2 medium; however, the optimal PGR combination that provided the highest shoot regeneration percentage and number of shoots per explant varied depending on the cultivars used. The shoots obtained from the Type 3 medium were able to transform to normal plants, and the normalized plants survived under greenhouse conditions. Moreover, according to the results of RAPD analysis, the normalized plants were genetically stable and similar to in vitro grown normal plants. Therefore, these results suggest that regeneration of shoots from vitrified leaves is possible for cultivars in which normal leaves are unable to regenerate shoots, although this depends on the type of vitrified leaves. However, as the shoot regeneration percentage and number of shoots per explant obtained in this study were still low, continued investigation is required for efficient propagation and genetic transformation of recalcitrant carnation cultivars

## 4. Materials and Methods

### 4.1. Plant Materials

Three different cultivars of in vitro grown carnations (*D. caryophyllus* L.), ‘Kumbuyl’ (KB), ‘Denev’ (DNV), and ‘Jinju’, were provided by Gyeongsang National University. The plants were cultured on hormone-free Murashige and Skoog (MS) basal medium and retained at 25 °C ± 2 °C under a 16 h photoperiod with 70 µmoL m^−2^s^−1^ light intensity for 2 weeks.

### 4.2. Induction of Normal and Vitrified Leaves Development

To test the shoot induction efficiency of normal and vitrified leaves, normal shoots were produced by culturing leaves on Type 1 medium, while vitrified shoots were produced by culturing leaves on Type 2 and Type 3 media for all cultivars (Table 2). The culture bottles were placed at 25 °C ± 2 °C under a 16 h photoperiod with 70 µmoL m^−2^s^−1^ light intensity. After four weeks of culture, the resulting shoots were sub-cultured every two weeks, and their leaves were used as explant sources for shoot induction.

### 4.3. Shoot Induction from Normal and Vitrified Leaves

Normal and vitrified leaves of eight-week-old in vitro shoots of the three different cultivars (KB, DNV, and Jinju) were transversely cut into small segments (approximately 0.5–1.0 cm). The leaf segments were cultured on MS medium supplemented with different concentrations of thidiazuron (TDZ) and 3-indole butyric acid (IBA) (Table 2), 3% sucrose, 0.8% agar, and pH 5.8. The culture was then incubated at 25 ± 2 °C with a 16 h photoperiod with 70 µmol m^−2^s^−1^ light intensity. Each treatment contained ten explants with three replicates. After four weeks of culture, the percentage of explants with shoots and number of shoots per explant were evaluated.

### 4.4. Plant Growth and Rooting

Thirty vitrified shoots per cultivar (approximately 1–1.5 cm in height) obtained from the above experiment were transplanted into hormone-free MS medium (containing 1.0% *w/v* agar) for plant growth and rooting. The culture bottles were placed at 25 °C ± 2 °C under a 16 h photoperiod with 70 µmoL m^−2^s^−1^ light intensity. After four weeks of culture, we investigated whether the vitrified shoots were converted to normal shoots. Normalized shoots were subsequently transferred to the same media two or three times to obtain complete normalized plants.

### 4.5. Acclimatization of in vitro Regenerated Plants

Four-week-old in vitro rooted plantlets (30 plants per cultivar) were removed from their culture vessels and washed gently under running tap water to remove adhering medium. The plantlets were grown in small pots containing peat-based soil and covered with polyethylene bags to maintain humidity (90–100%). After one week, polyethylene bags were removed and the acclimatized plants were transferred to the greenhouse. Their survival rate was evaluated after four weeks of transplanting.

### 4.6. Detection of Genetic Variation by RAPD

Total genomic DNA was isolated from the leaves of vitrified leaf-induced normalized plants (ten plants per cultivar) and in vitro grown normal plants (five plants per cultivar) using a HiYield genomic DNA mini Kit (Real Biotech Corporation, Taipei, Taiwan). RAPD amplification was performed using the method described by Naing et al. [30]. The primers and PCR conditions used for analysis are described in Supplementary Table S1. The reaction products were analyzed via electrophoresis on a 2% (*w/v*) agarose gel stained with 0.01 % EcoDye^TM^ (Solgent co., Seoul, Korea) and photographed under UV light exposure. RAPD analysis using each primer was repeated for five samples (in vitro grown normal plants) and ten samples (vitrified leaf-induced normalized plants) each for all cultivars to verify the banding pattern of the studied DNA samples. Analysis was repeated at least three times for all samples.

### 4.7. Statistical Analysis

Experimental data were statistically analyzed using SPSS version 25 software (IBM) and one-way analysis of variance. Data are represented as the mean of three replications, and means with the same letters are not significant using the Duncan’s Multiple Range Test (DMRT, *p* < 0.05).

## Figures and Tables

**Table 1:** Effect of different concentrations of plant growth regulators (PGR) combinations on shoot induction from Type 3 medium-derived vitrified leaf explants.

Cultivars	Plant Growth Regulators (mg/L)		Shoot Induction (%) *	Mean Number of Shoots/Explants
	IBA	TDZ		
	0	0	0.00 ^f^	0.00 ^e^
Jinju	0.1	0.2	66.67 ^a^	3.46 ^a^
	0.3	0.2	6.67 ^e^	4.33 ^a^
	0.5	0.2	13.33 ^e^	1.67 ^c^
	1.0	0.2	16.67^de^	1.83 ^c^
	2.0	0.2	10.00 ^e^	1.33 ^c^
	0.5	0.1	53.33 ^b^	1.40 ^c^
	0.5	0.3	20.00 ^cd^	3.89 ^a^
	0.5	0.5	26.67 ^cd^	2.53 ^b^
	0.5	1.0	30.00 ^c^	0.64 ^d^
	0.5	2.0	20.00 ^cd^	3.33 ^ab^
DNV	0	0	0.00 ^d^	0.00 ^e^
	0.1	0.2	10.00 ^bc^	3.33 ^ab^
	0.3	0.2	6.67 ^bc^	3.33 ^ab^
	0.5	0.2	3.33 ^c^	0.67 ^d^
	1.0	0.2	16.67 ^bc^	3.83 ^a^
	2.0	0.2	13.33 ^bc^	1.50 ^c^
	0.5	0.1	10.00 ^bc^	1.67 ^c^
	0.5	0.3	26.67 ^a^	2.83 ^b^
	0.5	0.5	16.67 ^bc^	1.67 ^c^
	0.5	1.0	16.67 ^bc^	2.25 ^b^
	0.5	2.0	13.33 ^bc^	1.67 ^c^
KB	0	0	0.00 ^d^	0.00 ^d^
	0.1	0.2	26.67 ^a^	2.90 ^b^
	0.3	0.2	3.33 ^c^	0.67 ^c^
	0.5	0.2	6.67 ^bc^	0.67 ^c^
	1.0	0.2	3.33 ^c^	0.67 ^c^
	2.0	0.2	3.33 ^c^	0.67 ^c^
	0.5	0.1	13.33 ^bc^	5.00 ^a^
	0.5	0.3	3.33 ^c^	0.33 ^d^
	0.5	0.5	3.33 ^c^	2.00 ^b^
	0.5	1.0	6.67 ^b^	0.67 ^c^
	0.5	2.0	26.67 ^a^	0.50 ^c^

* Percent of explants forming shoots. Data are represented as the mean of three replicates per treatment. Means with the same letter within a column are not significantly different according to the Duncan’s multiple range test (DMRT, *p* > 0.05). Analysis was performed separately for each cultivar.

**Table 2:** Components of the three types of media used for the induction of normal and vitrified leaves development.

Name of Medium	Components
Type 1	MS basal medium [34], 3% sucrose, 0.8% plant agar, pH 5.8
Type 2	MS Gamborg B5 vitamins medium [35], 2-morpholinoethanesulfonic acid(MES) monohydrate 590 mg/L, BA 0.5 mg/L, NAA 0.02 mg/L, 3% sucrose, 0.25% gelrite, pH 5.8
Type 3	MS medium, adenine 80 mg/L, sodium phosphate monobasic (NaH_2_PO_4_) 85 mg/L, 2% sucrose, 0.8% plant agar, pH 5.7

## Supplementary Materials

The following are available online at https://www.mdpi.com/2223-7747/9/8/950/s1, Table S1: Primers, sequences, and PCR conditions used for RAPD analysis.
