# Supplementary material for: Regeneration of Genetically Stable Plants from in Vitro Vitrified Leaves of Different Carnation Cultivars
Source: Plants (Basel). 2020 Jul 28;9(8):950. doi: 10.3390/plants9080950 (PMC7465169; doi:10.3390/plants9080950)
Supplement: Supplementary file 1 [file plants-09-00950-s001.pdf]

Supplementary Files

Table S1. Primers, sequences, and PCR conditions used for RAPD analysis

Primers	Sequence	Temperature (℃)
OPA-01	CAG GCC CTT C	33
OPA-02	TGC CGA GCT G	33
OPA-03	AGT CAG CCA C	28.9
OPA-04	AAT CGG GCT G	28.9
OPA-05	AGG GGT CTT G	28.9
OPA-06	GGT CCC TGA C	33
OPA-07	GAA ACG GGT G	28.9
OPA-08	GTG ACG TAG G	28.9
OPA-09	GGG TAA CGC C	33
OPA-10	GTG ATC GCA G	28.9
OPA-11	CAA TCG CCG T	28.9
OPA-12	TCG GCG ATA G	28.9
OPA-13	CAG CAC CCA C	33
OPA-14	TCT GTG CTG G	28.9
OPA-15	TTC CGA ACC C	28.9
OPA-16	AGC CAG CGA A	28.9
OPA-17	GAC CGC TTG T	28.9
OPA-18	AGG TGA CCG T	28.9
OPA-19	CAA ACG TCG G	28.9
OPA-20	GTT GCA ATC C	28.9
OPB-01	GTT TCG CTC C	28.9
OPB-02	TGA TCC CTG G	28.9
OPB-03	CAT CCC CCT G	33
OPB-04	GGA CTG GAG T	28.9
OPB-05	TGC GCC CTT C	33
OPB-06	TGC TCT GCC C	33
OPB-07	GGT GAC GCA G	33
OPB-08	GTC CAC ACG G	33
OPB-09	TGG GGG ACT C	33
OPB-10	CTG CTG GGA C	33
OPB-11	GTA GAC CCG T	28.9
OPB-12	CCT TGA CGC A	28.9
OPB-13	TTC CCC CGC T	33
OPB-14	TCC GCT CTG G	33
OPB-15	GGA GGG TGT T	28.9
OPB-16	TTT GCC CGG A	28.9
OPB-17	AGG GAA CGA G	28.9
OPB-18	CCA CAG CAG T	28.9
OPB-19	ACC CCC GAA G	33
OPB-20	GGA CCC TTA C	28.9

PCR condition: 95 ℃ for 2min, followed by 35 cycles of 95 ℃ 20 s, Temperature (℃) 53s, 72 ℃ for 1 min and 72 ℃ 5 min
